# The contribution of the cable to the polarity effect in ionization chamber dosimetry

**DOI:** 10.1002/acm2.70327

**Published:** 2025-11-21

**Authors:** Duncan J. Butler, Brendan J. Healy

**Affiliations:** ^1^ Medical Radiation Services Australian Radiation Protection and Nuclear Safety Agency Yallambie Victoria Australia; ^2^ Peter MacCallum Cancer Centre Bendigo Victoria Australia

**Keywords:** cable effect, extra‐cameral signal, ionization chamber, polarity effect

## Abstract

**Background:**

The polarity effect refers to the difference in the magnitude of the electrical signal produced by an ionization chamber at opposite bias voltages. There are multiple causes, one of which is the small electrical current produced in parts of the ionization chamber, including the electrical connections, produced directly by radiation.

**Purpose:**

To show that cable irradiation can be the main source of anomalously large polarity effects in certain cases when small volume chambers are used in large megavoltage radiation fields. To present an alternative to the use of a multiplicative polarity correction.

**Methods:**

The polarity correction for a small volume (0.02 cm^3^) ionization chamber was measured at 1.5 cm depth in a variety of field sizes in a 36 cm x 32 cm x 30 cm water tank for 6 MV photon and 6 MeV electron beams. Depth‐ionization measurements were then made with the 25 cm x 25 cm field. Readings were taken at opposite bias voltages at each depth before moving to the next depth.

**Results:**

The polarity effect increased with field‐size in both cases. In the photon case the polarity correction increased from 0.5% to 2.0% over the depth range where charged particle equilibrium exists. In the electron case the polarity correction increased to very large values (∼400%) where the signal approached zero. The bias‐independent component was relatively constant at all depths. Measurements of the response of the cable without the chamber confirmed that it responds to scattered radiation.

**Conclusions:**

In our measurements the relatively large polarity corrections are due to a small bias‐independent contribution which arises in the cable. In these cases, the large polarity correction is a result of the definition of the correction as a relative factor, and not the result of anomalous behaviour in the ionization volume.

## 1 INTRODUCTION

Reversing the bias voltage on an ionization chamber results in an electrical current that is opposite in sign but usually slightly different in magnitude.^1^ The difference in magnitude is known as the polarity effect. Attix^2^ contains a list of seven possible causes, including the current produced directly by irradiation of the chamber and the cable, known as the “Compton current”.^1^, ^3^ The details of the production of this current are beyond the scope of our paper, but we refer to the explanation provided by Looe et al.,^4^ that is, there can be an imbalance between the secondary electrons ejected from and stopped within the chamber collecting electrode, including a contribution from the stem and cable.

That cable irradiation should be considered is well known in medical physics. For example Aget and Rosenwald^5^ showed that stem irradiation was responsible for the polarity effect observed in large field MeV electron beams, and Das et al.^6^ and Fiorino et al.^7^ recorded cable effects in large electron fields used for total body irradiation. That small chambers may exhibit large polarity effects is also well known, and best practice includes checking for this effect. See for example section III.A1 in AAPM TG‐106.^8^

There are multiple causes of the polarity correction in the chamber itself, see for example Kim et al ^9^ regarding thimble chambers, and Kron et al ^10^ and Abdel‐Rahman et al ^11^ regarding plane‐parallel chambers. Recently, Looe et al ^4^ showed using modelling and confirmed with measurements using different sized fields that the polarity effect can be explained by charge deposited/removed by radiation in the ionization chamber electrodes, including the stem and cable.

Several recent publications report large polarity corrections for which the cause is not determined.^12^, ^13^, ^14^ We do not have enough information to reproduce the setups in these cases: the cable positions, lengths, construction and the scattered radiation can be unique to any measurement. Instead, we manufacture situations where the polarity correction is large and show that the effect is probably due to cable irradiation. We present a simple analysis which, unlike the use of a multiplicative correction, works in situations where the response of the cable is of the same order of magnitude as the ionization current. We provide notes on situations where large corrections are expected and suggest data that should be reported when large corrections are encountered.

## 2 THEORY

We use *M*_+_ to refer to the reading on the electrometer at one polarity (“+”). This reading may be positive or negative and “+” may refer to either polarity (we note that electrometer models are not consistent in the polarity, in any case). We define *M*_‐_ as the reading, including the sign, at the opposite but otherwise equal bias voltage.

If we assume that the reading is the sum of a bias‐dependent component (most likely due to ionization in the active volume) *M_ion_* and a bias‐independent component *M_C_* (including Compton components produced in the chamber, stem, connectors and/or cables) then:

(1) $$\;M_{+}=M_{ion}+M_{C}$$

(2) $$\;M_{-}=-M_{ion}+M_{C}$$

Here *M*_+_ and *M*_‐_ may take positive, zero or negative values. By definition *M_ion_* and *M_C_* have the same value at both bias voltages. They can also both take positive, zero or negative values.

It follows from (1) and (2) that

(3) $$M_{\mathrm{ion}}=(M_{+}\;-M_{-})/2$$

(4) $$M_{C}=\left(M_{+}+M_{\_}\right)/2$$

We note that we have used *M* (for electrometer reading) but the formulae are the same for ionization currents *I* and charges *Q*. The quantities are corrected for background, leakage and the electrometer calibration factor. For dosimetry, the signal from the chamber is assumed to be |*M_ion_* |.

In dosimetry for radiotherapy, it is common practice to define a multiplicative correction to apply to the electrometer reading to arrive at the average magnitude of the signal at opposite polarities. TRS‐398^15^ defines *k*_pol_ for the normal operating bias. We use their definition and define *k*_pol_ for both polarities:

(5) $$\;k_{pol,-}=\frac{\left|M_{+}\right|+\left|M_{-}\right|}{2\left|M_{-}\right|}\;$$

(6) $$\;k_{pol,+}=\frac{\left|M_{+}\right|+\left|M_{-}\right|}{2\left|M_{+}\right|}\;$$

Note that these factors are positive: *k*_pol,‐_ would multiply the absolute value of the reading |*M_‐_* | and *k*_pol,+_ would multiply |*M_+_* | to obtain the average magnitude of the ionization current measured at each bias. The formulism is intended for the case when |*M_ion_* | is larger than |*M_C_* |, which is true for nearly all radiotherapy measurements.

## 3 METHOD

**FIGURE 1:**
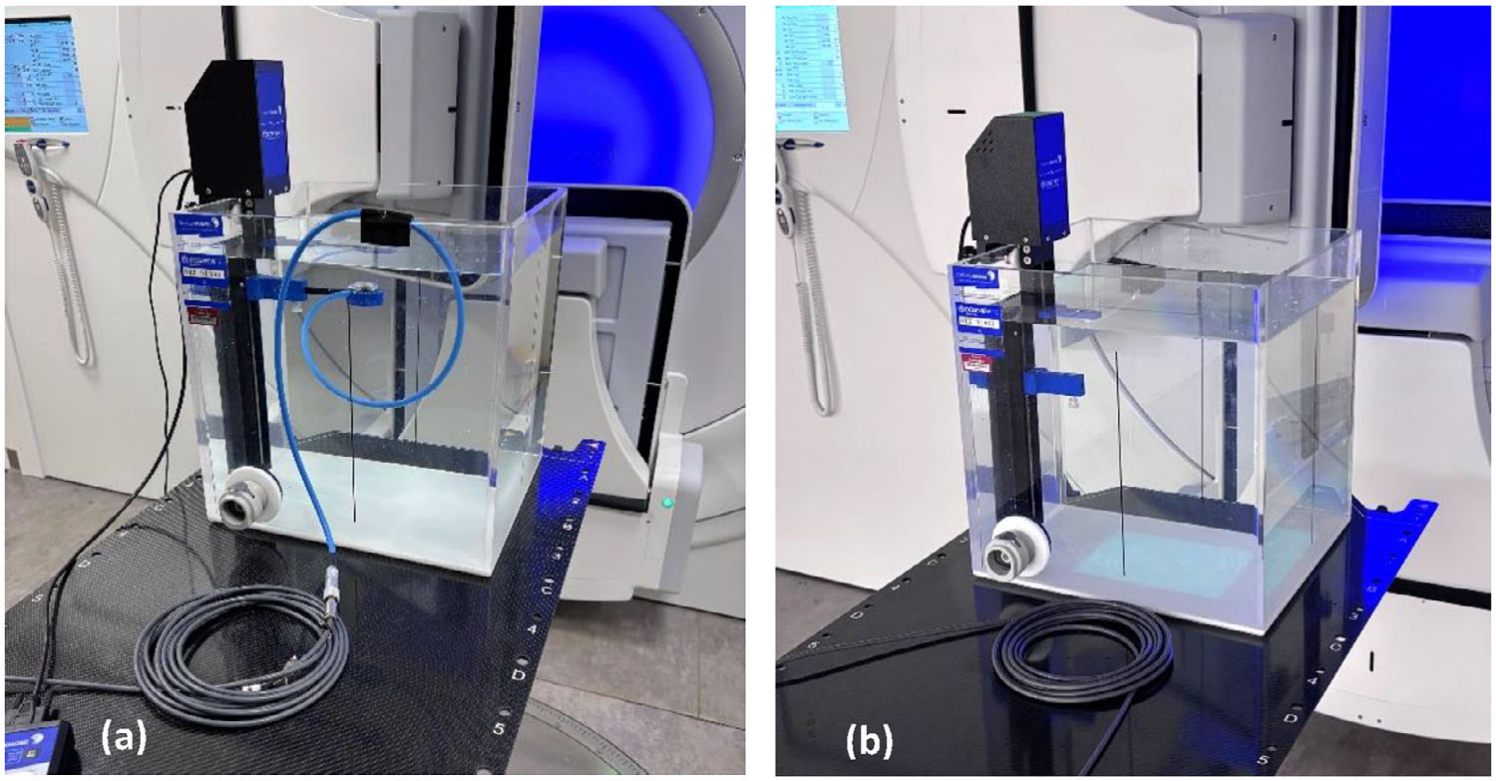
(a) Setup of Advanced Markus chamber in water tank, and (b) cable next to water tank with no ionization chamber.

To investigate the role of the cable, we measured the polarity correction in linear accelerator electron and photon beams using a very small volume chamber in conjunction with large field sizes. We used a 36 cm x 32 cm x 30 cm water tank and placed some 5 m of extension cable on the couch top next to the phantom (Figure 1a). The chamber cable was draped over the lip of the tank and connected to the extension on the couch. A second arrangement consisted of only an extension chamber without a chamber attached (Figure 1b). The investigations are summarized in Table 1.

**TABLE 1:** Measurements used to investigate the role of the cable in the polarity effect.

Detector	Case	Measurement
PTW model 34045 Advanced Markus (cap on)	2.1	Field size dependence at 1.5 cm depth in water for 6 MV photon and 6 MeV electron beams
	2.2	6 MV photon depth‐ionization in 25 cm x 25 cm field in water
	2.3	6 MeV electron depth‐ionization in 25 cm x 25 cm field (without applicator) in water
	2.4	Reproducibility
No chamber: cable end shielded and placed away from source	2.5	Field‐size dependence of cable signal for 6 MV photon and 6 MeV electron beams

The ionization chamber was a small volume plane‐parallel chamber (PTW model 34045 Advanced Markus chamber, collecting volume 0.02 cm^3^) operated at ‐300 V and +300 V. Some 4 m of extension cable was coiled on the couch next to the phantom (Figure 1a). The electrometer was a PTW Model T10022 “Webline” on the low range. We used *M*_‐_ to refer to the electrometer setting of ‐300 V and *M*_+_ to the opposite bias, +300 V, where setting ‐300 V results in the central pin of the cable and the collecting electrode of the ionization chamber being positive. We refer to this polarity as CEP.

The linac was an Elekta Versa and the water tank was a Sun Nuclear Doseview 1D. The tank was filled with water to create a volume approximately 36 cm x 32 cm x 30 cm (high) and positioned with 100 cm from the source to the water surface. Irradiations consisted of 200 MU where the linac was calibrated for 1 cGy/MU for the reference depth and settings in Table 2. The chamber was positioned with the outside top of the buildup cap level with the water surface. The inner surface of the front window, which we took to be the reference point of the chamber, is then at a depth of 1 mm.

**TABLE 2:** Beam parameters and reference conditions for the Elekta Versa photon and electron beams. The linac is calibrated to deliver 1 cGy/MU at the reference depth *z*_ref_ for photons, at the depth of maximum dose, *d*_max_, for electrons. MU = Monitor Unit, a unit of the linac's internal ionization chamber.

Beam identifier	Beam quality *TPR*_20,10_ or *R*_50_	Source‐surface distance cm	Reference depth, *z_ref_* cm	MU calibration coefficient^a^ at *z*_ref_ cGy/MU	Linac output rate MU/min
6 MV	0.684	90	10	1	400
10 MV	0.733	90	10	1	380
18 MV	0.779	90	10	1	470
6 E	2.45 cm	100	1.37	0.997	600
9 E	3.56 cm	100	2.04	0.999	600
12 E	4.70 cm	100	2.72	0.998	600
15 E	5.89 cm	100	3.43	0.986	600

^a^ For the 10 cm x 10 cm field‐size for photons, 10 cm x 10 cm applicator for electrons.

### 3.1 Field‐size dependence

The reference point of the chamber was set to a depth of 16 mm and the field‐size varied from 2.5 cm x 2.5 cm to 34 cm x 34 cm using the Multi Leaf Collimator (MLC). The charge was measured at each polarity (± 300 V) for both the 6 MV photon beam and 6 MeV electron beam. For electrons, an applicator was not used, and the field size was defined by the MLC which was positioned as for the corresponding photon field‐size. To illustrate the lateral extent of these fields, the profiles were measured with a Sun Nuclear IC Profiler at 16 mm depth.

### 3.2 Photon depth‐ionization

Depth‐ionization curves (PDI's) were measured for the 25 cm x 25 cm field for two polarities in the 6 MV photon beam. The PDI's were measured by setting the depth, then measuring the charge produced at each polarity for 200 MU before moving to the next depth. This approach reduces the possibility of the cable being in a different location because of chamber motion in the tank. Measurements were also made with the chamber out of the water (negative depths). Temperature and pressure readings were taken to ensure constancy but were not used to correct the charge readings. The sign of the charge is as recorded on the electrometer.

### 3.3 Electron depth‐ionization

The PDI was repeated but for the 25 cm x 25 cm 6 MeV electron field. As the electrons do not penetrate as far, a much smaller depth range was measured: some 5 cm instead of 25 cm for the 6 MV photon beam.

### 3.4 Reproducibility

The reproducibility of the charge measurements in Section 2.3 was assessed by repeating charge measurements while (a) the chamber remained motionless, and (b) the chamber was moved in the tank but returned to the same position. Then entire depth‐ionization measurement was repeated (c) setting up the tank and cable from scratch, and (d) setting up from scratch but attempting to reproduce the cable geometry (same extension cable, length on couch and position).

### 3.5 Cable response to scattered beam

Finally, we measured the charge arising from the cable without an ionization chamber. Instead, a length of some 4 m of cable was coiled up on the couch next to the water tank (Figure 1b). To ensure there was no response from the end of the cable (which can act as an ionization chamber) it was positioned far from the water tank and shielded with Pb. Charge measurements were made for 6 MV photons and 6 MeV electrons for various field sizes. This measurement was also performed with a solid water stack for all the available beam energies by recording only the electrical current with zero bias voltage.

## 4 RESULTS

### 4.1 Field‐size dependence

**FIGURE 2:**
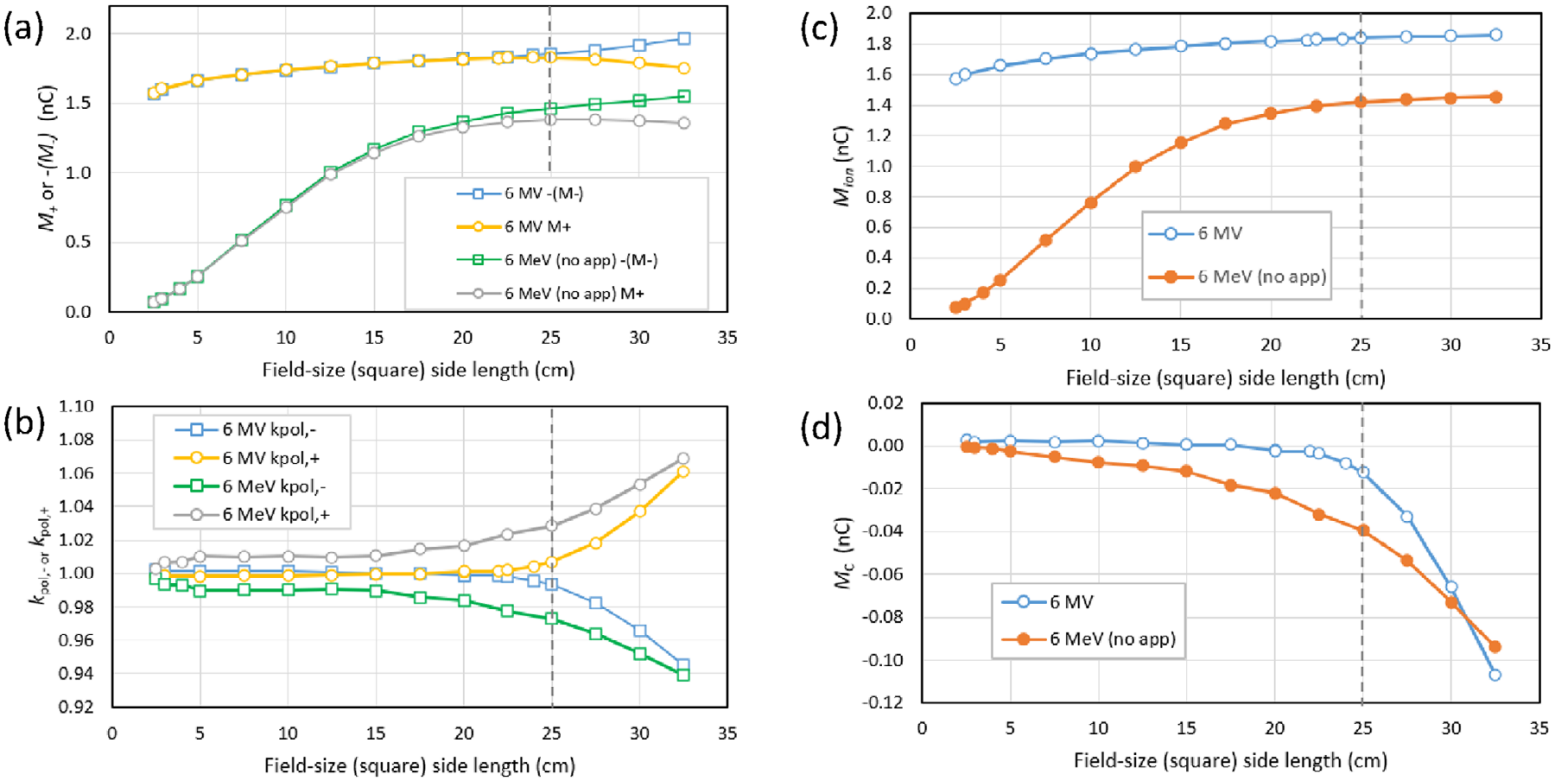
(a) Field size dependence of the signal for 6 MV photon and 6 MeV electron beam with MLC‐defined field size (no applicator) with chamber cavity at 1.6 cm depth, (b) the polarity correction, (c) the bias‐dependent component *M*_ion_ and (d) the bias‐independent component *M_C_*. The dashed line shows the field size chosen for depth‐ionization measurements (25 cm x 25 cm).

The field size dependence for both the 6 MV photon and 6 MeV electron beam is shown in Figure 2a. To aid the comparison of opposite bias voltages, the negative of *M*_‐_ is plotted. The corresponding polarity correction is shown in Figure 2b, while the bias‐dependent and bias‐independent components are in (c) and (d). We note that a field‐size of 24.6 cm at 1 m becomes 32 cm at the bottom of the tank (1.3 m from the source), equal to the width of the tank in the direction of the cable.

**FIGURE 3:**
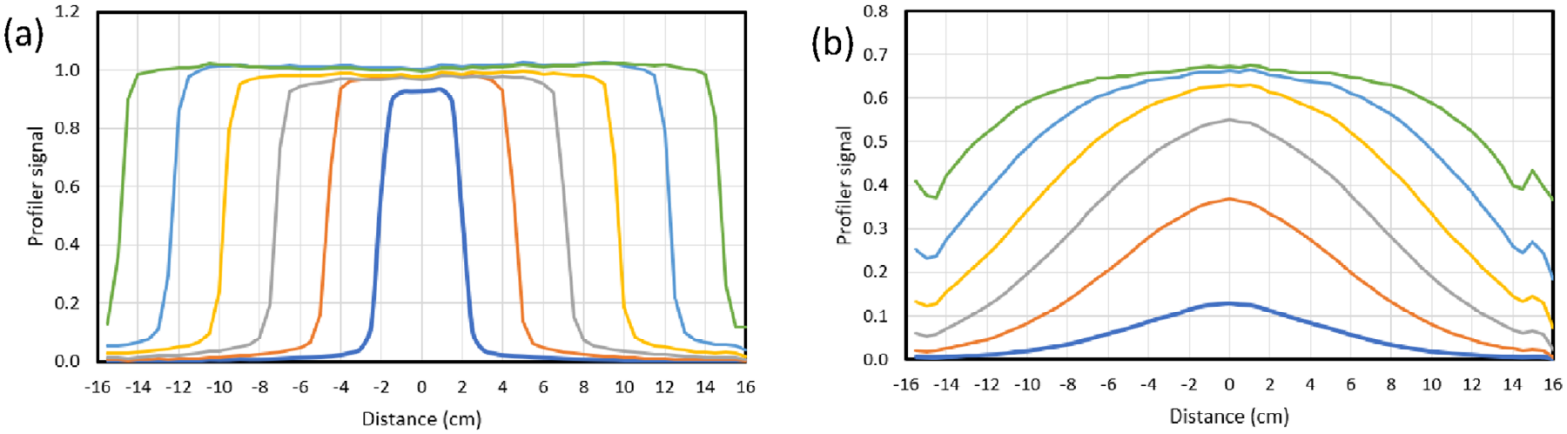
Crossline beam profiles at 1.6 cm depth obtained with an ionization chamber array (Sun Nuclear IC Profiler) for field sizes from 5 cm x 5 cm to 30 cm x 30 cm in increments of 5 cm. The measurements are normalised to 1 for the 30 cm x 30 cm 6 MV photon beam (a).

The lateral (crossline) dose profiles for 6 MV photons (a) and 6 MeV electrons (b) are shown in Figure 3. Note that without the applicator, the 6 MeV electron beam (b) extends well beyond the size of the water tank even for small field‐sizes.

### 4.2 Photon depth‐ionization

**FIGURE 4:**
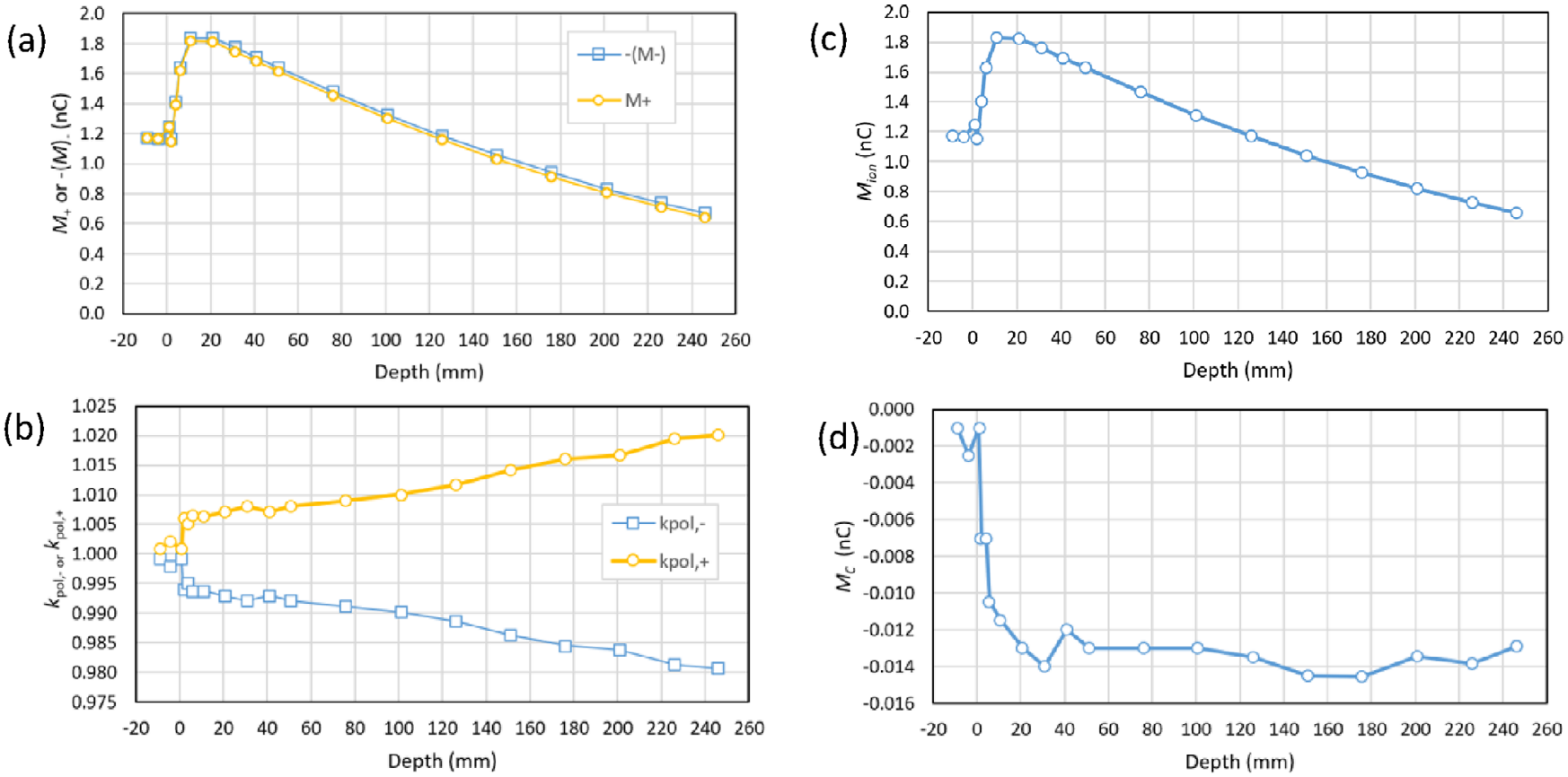
(a) 6 MV linac photon depth‐ionisation curve measured with an Advanced Markus chamber, (b) the polarity correction, (c) the bias‐dependent component *M*_ion_, and (d) the bias‐independent component *M_C_*. The reproducibility of *M*_C_ was some 0.002 nC but see the discussion in Section 3.4.

The photon depth‐ionization measurements are given in Table 3 along with the bias‐dependent component *M_ion_* and bias‐independent component *M_C_*. Figure 4(a) shows the depth‐ionisation curve as measured for each polarity, where −(*M*_‐_) has been plotted to aid in the comparison with *M_+_*. Figure 4(b) shows the polarity correction as a function of depth. Figure 4(c) shows the magnitude |*M_ion_* | and Figure 4(d) shows the bias‐independent component *M_C_*.

**TABLE 3:** Depth‐ionization curve obtained with an Advanced Markus chamber in a linac 6 MV photon beam. Charge produced for 200 MU delivered in approximately 27 s.

Depth mm	*M*_‐_ ^a^ nC	*M*_+_ nC	*‐(M*_‐_) nC	*M_ion_* nC	*M_C_* nC	*k*_pol,‐_	*k*_pol,+_
−9.0	−1.172	1.170	1.172	1.171	−0.001	0.999	1.001
−4.0	−1.167	1.162	1.167	1.165	−0.003	0.998	1.002
1.0	−1.247	1.245	1.247	1.246	−0.001	0.999	1.001
2.0	−1.162	1.148	1.162	1.155	−0.007	0.994	1.006
4.0	−1.409	1.395	1.409	1.402	−0.007	0.995	1.005
6.0	−1.640	1.619	1.640	1.630	−0.011	0.994	1.006
11.0	−1.839	1.816	1.839	1.828	−0.012	0.994	1.006
21.0	−1.838	1.812	1.838	1.825	−0.013	0.993	1.007
31.0	−1.774	1.746	1.774	1.760	−0.014	0.992	1.008
41.0	−1.706	1.682	1.706	1.694	−0.012	0.993	1.007
51.0	−1.640	1.614	1.640	1.627	−0.013	0.992	1.008
76.0	−1.480	1.454	1.480	1.467	−0.013	0.991	1.009
101.0	−1.325	1.299	1.325	1.312	−0.013	0.990	1.010
126.0	−1.185	1.158	1.185	1.172	−0.014	0.989	1.012
151.0	−1.056	1.027	1.056	1.042	−0.015	0.986	1.014
176.0	−0.940	0.911	0.940	0.926	−0.015	0.985	1.016
201.0	−0.834	0.807	0.834	0.820	−0.013	0.984	1.017
226.0	−0.739	0.711	0.739	0.725	−0.014	0.981	1.019
246.0	−0.668	0.643	0.668	0.656	−0.013	0.981	1.020

^a^ The electrometer was set to ‐300 V (CEP).

### 4.3 Electron depth‐ionization

**FIGURE 5:**
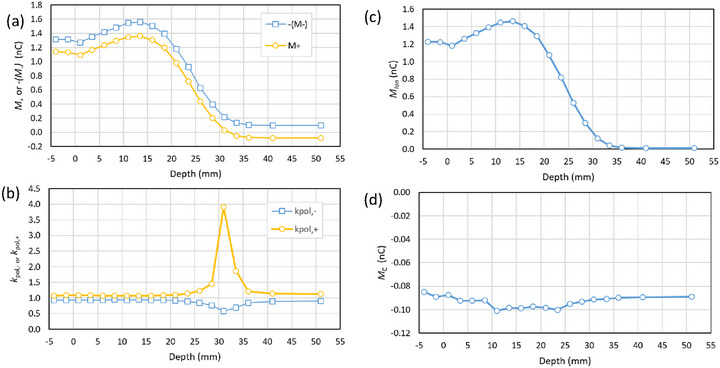
(a) 6 MeV linac electron depth‐ionisation curve measured with an Advanced Markus chamber, (b) the polarity correction, (c) the bias‐dependent component *M*_ion_, and (d) the bias‐independent component *M_C_*.

The electron depth‐ionization measurements are given in Table 4 along with the calculated polarity correction and bias‐independent current. Figure 5 shows the same quantities as Figure 4, but for the electron beam (See Table 4 ).

**TABLE 4:** Depth‐ionization curve obtained with an Advanced Markus chamber in a linac 6 MeV electron beam. Charge produced for 200 MU delivered in approximately 20 s.

Depth mm	*M*_‐_ nC	*M*_+_ nC	*‐(M*_‐_) nC	*M_ion_* nC	*M_C_* nC	*k*_pol,‐_	*k*_pol,+_
−4.0	−1.310	1.140	1.310	1.225	−0.085	0.935	1.075
−1.5	−1.311	1.133	1.311	1.222	−0.089	0.932	1.079
1.0	−1.267	1.092	1.267	1.180	−0.087	0.931	1.080
3.5	−1.352	1.167	1.352	1.260	−0.093	0.932	1.079
6.0	−1.417	1.232	1.417	1.325	−0.093	0.935	1.075
8.5	−1.481	1.297	1.481	1.389	−0.092	0.938	1.071
11.0	−1.548	1.346	1.548	1.447	−0.101	0.935	1.075
13.5	−1.557	1.360	1.557	1.459	−0.098	0.937	1.072
16.0	−1.503	1.305	1.503	1.404	−0.099	0.934	1.076
18.5	−1.390	1.195	1.390	1.293	−0.097	0.930	1.082
21.0	−1.175	0.978	1.175	1.077	−0.099	0.916	1.101
23.5	−0.922	0.721	0.922	0.821	−0.100	0.891	1.139
26.0	−0.625	0.435	0.625	0.530	−0.095	0.848	1.219
28.5	−0.391	0.205	0.391	0.298	−0.093	0.762	1.455
31.0	−0.214	0.031	0.214	0.123	−0.091	0.573	3.909
33.5	−0.133	−0.049	0.133	0.042	−0.091	0.683	1.864
36.0	−0.106	−0.074	0.106	0.016	−0.090	0.850	1.214
41.0	−0.100	−0.078	0.100	0.011	−0.089	0.891	1.139
51.0	−0.099	−0.079	0.099	0.010	−0.089	0.896	1.131

### 4.4 Reproducibility

**FIGURE 6:**
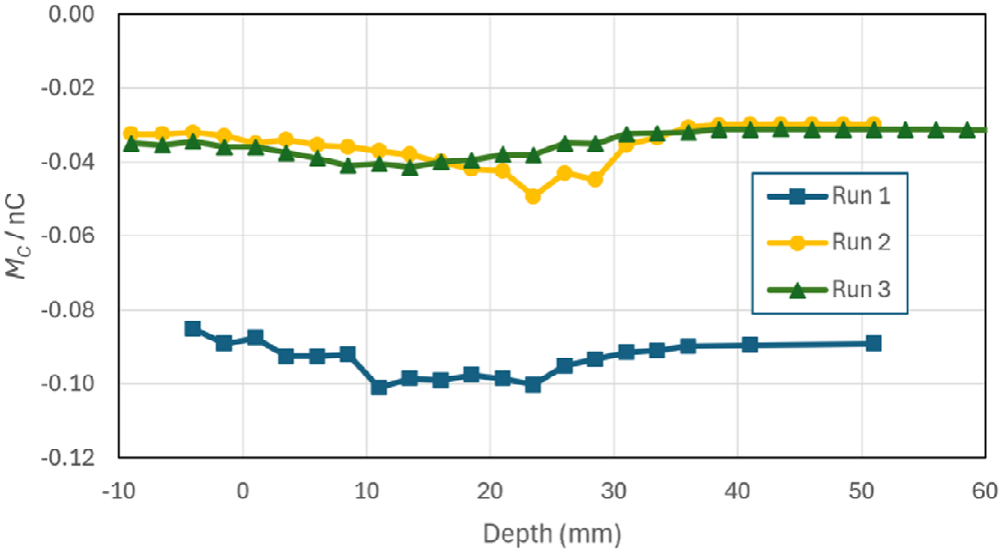
The component *M_C_* from three different determinations of the 6 MeV electron PDI. Run 1 is from Figure 4. Runs 2 and 3 were set up on another linac but with an attempt to reproduce the cable positions between the runs.

The short‐term reproducibility of each charge measurement at each depth was better than 0.002 nC. Figure 6 shows a comparison of three repeat measurements of the 6 MeV 25 cm x 25 cm field. Run 1 shows the data from Figure 5(d). Runs 2 and 3 were independent setups on a different linac. Runs 2 and 3 were set up from scratch but an attempt was made to position the same length of cable in the same place. In this case, *M*_C_ was repeatable to within 0.01 nC.

### 4.5 Cable response to scattered beam

**FIGURE 7:**
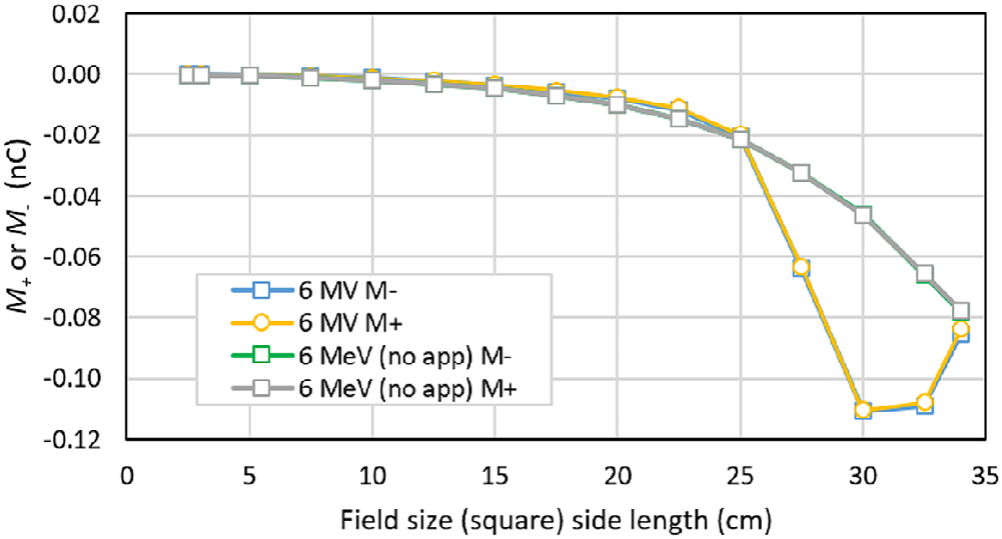
Charge produced at opposite polarities with the cable coiled up next to the tank, and no ionization chamber attached.

With no ionization chamber attached and the cable coiled next to the water phantom, the charge produced for each polarity is plotted in Figure 7 for both the 6 MV photon and 6 MeV electron beam.

**FIGURE 8:**
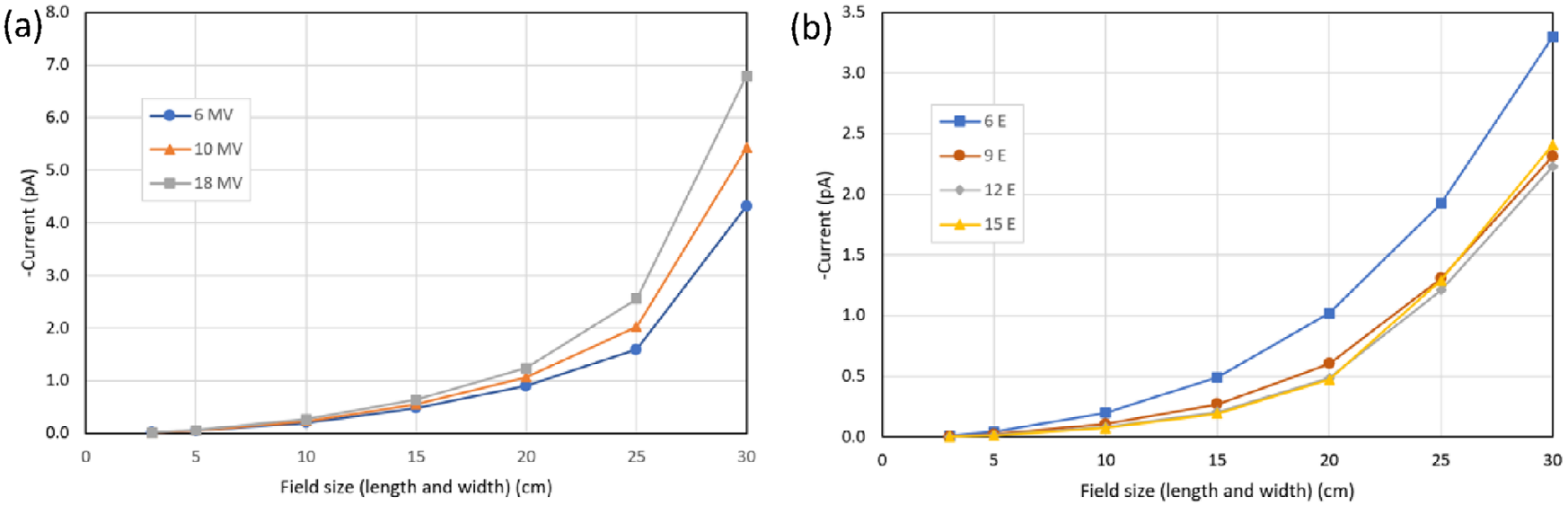
Electrical current produced by some 5 m of triaxial cable coiled up on the couch adjacent to a stack of solid water (30 cm x 30 cm x 30 cm) for (a) linac photon beams and (b) linac electron beams (without applicator and MLC used to set field‐size). In all cases the currents were negative.

Figure 8 shows the magnitude of the electrical current measured at zero bias for all of the available beams.

## 5 DISCUSSION

### 5.1 Field‐size dependence

As the field size increases, for both the electron and photon beams the magnitudes of *M*_+_ and *M*_‐_ diverge (Figure 2a). As a result, the polarity correction deviates further from unity for larger fields (Figure 2b). In the photon beam the bias‐dependent component increases relatively slowly (Figure 2c), while for the electron beam it has a very low value on the central axis and increases rapidly. In both cases the bias‐independent component increases in magnitude with field size. We interpret these results as evidence that the bias‐independence component *M*_C_ arises predominately in the cable. In particular, once the field is larger than the water tank *M*_+_ and *M*_‐_ diverge more strongly, which we would not expect if *M*_C_ was coming from the ionization chamber.

### 5.2 Photon depth‐ionization

At or above the surface the photon measurements show that the polarity correction is near unity. Once submerged, the magnitude of the polarity correction increases with depth from some 0.5% near the surface to 2% at 25 cm depth, Figure 4b. We do not have an explanation for the small (i.e., unity) polarity correction at the surface. Polarity corrections as high as 12% have been reported for the Advanced Markus when used in solid water without its buildup cap.^12^ We can only note that our chamber had 1 mm of buildup (the waterproof cap) at all times and suggest that small changes in the signal from the chamber or the cable as they exit the water are responsible for the sudden change seen at that point. However, as discussed in Section 4.6, isolating the precise origin of these small signals is difficult.

The thrust of our argument concerns the behaviour of the polarity correction with depth. Clearly the correction increases with depth, however it can be seen in Figure 4d that the bias‐independent component is relatively constant beyond the depth of maximum dose. The only change is that the signal from the chamber is smaller, and as the polarity correction is a relative factor, it increases.

### 5.3 Electron depth‐ionization

The electron depth ionization measurements in Figure 5 show a bias‐independent component *M_C_* of some ‐0.09 nC in 200 MU compared to ‐0.014 nC for the photon case. The difference could be due to the different linac beam profiles (Figure 3) and/or spectra of the two beams. It could also be due to differences in the cable geometry as the exact position and length of cable was not reproduced on the two days of measurement. Two additional measurements gave values nearer to ‐0.04 nC (Section 4.4). We note that the electron beam without the applicator is extremely broad, and a considerable part of the cable was irradiated. A measurement at a single depth with a 25 cm x 25 cm applicator on a different linac yielded a value of ‐0.02 nC. Interestingly, the curve does not exhibit a change in behaviour when the chamber is at or above the surface.

Unlike the photon beam, where the maximum depth of 250 mm resulted in a dose reduction of only 30%, the electron depth dose falls to very low values as we reach the practical range of the electron beam, some 30 mm. Hence the polarity correction takes anomalously high values (Figure 5b—up to nearly 400%) and would have been undefined if one of the readings *M*_+_ had been exactly zero. This point on the graph is where *M_+_* changes sign and the curve crosses the axis at around 33 mm depth, where *M*_ion_ is equal to ‐*M_C_*. In this case the analysis using Equations (3) and (4) is more amenable than that of Equations (5) and (6).

### 5.4 Reproducibility

The measurements were highly reproducible provided no change was made to the setup. A reproducibility of 0.002 nC in *M*_+_ and *M*_‐_ results in a reproducibility of some 0.0014 nC in *M*_C_ at most depths. However, once the depth of the chamber was adjusted, when returned to the same depth the signal reproducibility decreased. Figure 5 shows that with attempts to reproduce the cable position and tank setup the reproducibility was of the order 0.01 nC in *M*_C_. Nevertheless, qualitative aspects of both the photon and electrons depth‐ionization measurements were highly reproducible.

### 5.5 Cable response to scattered beam

The measurements in Figrue 7 show that the signal from the cable increases with field‐size. The bias‐dependent component *M*_ion_ was nearly zero for all cases (*M*_+_ = *M*_‐_). The value of the bias‐independent component *M*_C_ at 25 cm x 25 cm was ‐0.02 nC for both beams. This is consistent with values observed in the depth‐ionization measurements (−0.034 and −0.09 nC) noting that the cable in the depth‐ionization measurements was exposed to more radiation, being closer to the direct beam. The sudden change in the photon case (Figure 7a) at 25 cm x 25 cm is probably a result of the field‐size exceeding the tank size (in the electron case the fields are already larger than the tank—see the profiles in Figure 3).

**FIGURE 9:**
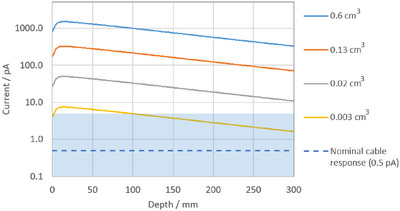
Approximate ionization current measured on the central axis during a depth‐dose measurement with a Farmer‐type chamber (0.6 cm^3^), a scanning chamber, e.g., CC13 (0.13 cm^3^), Advanced Markus or Pinpoint (0.02 cm^3^) and a micro chamber, e.g., IBA Razor Nano (0.003 cm^3^). The dotted line at 0.5 pA indicates a nominal value of the cable response, noting that in extreme cases it can be as large as 7 pA (the blue region) for very large fields. Here the ionization currents are calculated for 6 MV photons, 10 cm × 10 cm field, 500 cGy/min at *d*_max _= 1.4 cm.

Figure 8 shows electrical currents from the cable alone for all the linac beams. Here we show the magnitude of the current instead of the charge, for the discussion that follows. The magnitude of the currents is as high as 7 pA, with 0.5 pA being a more representative value. We note that extension cables and connectors are often the same dimensions regardless of the ionization chamber connected. In Figure 9 we show the nominal currents from ionization chambers of different volumes that might be available in the clinic in a typical depth‐dose measurement on a conventional medical linac and compare them to the typical cable currents we measured. We note that for Farmer‐type chambers the contribution from the cable is several orders of magnitude lower than the ionization current. However there can be situations where small volume chambers produce such small signals that the cable current is significant, and a large polarity effect is expected.

It is always possible to detect bias‐independent contributions, and |*M*_ion_| will result in an appropriate current or charge for dosimetry provided there is no bias‐dependent component arising in the stem or cable. We do not anticipate a risk of a clinical dosimetry error, provided recommendations to check for the polarity correction are observed. Simple steps such as not coiling up a large length of cable on the couch may also help.

### 5.6 Origin of radiation‐induced currents

**FIGURE 10:**
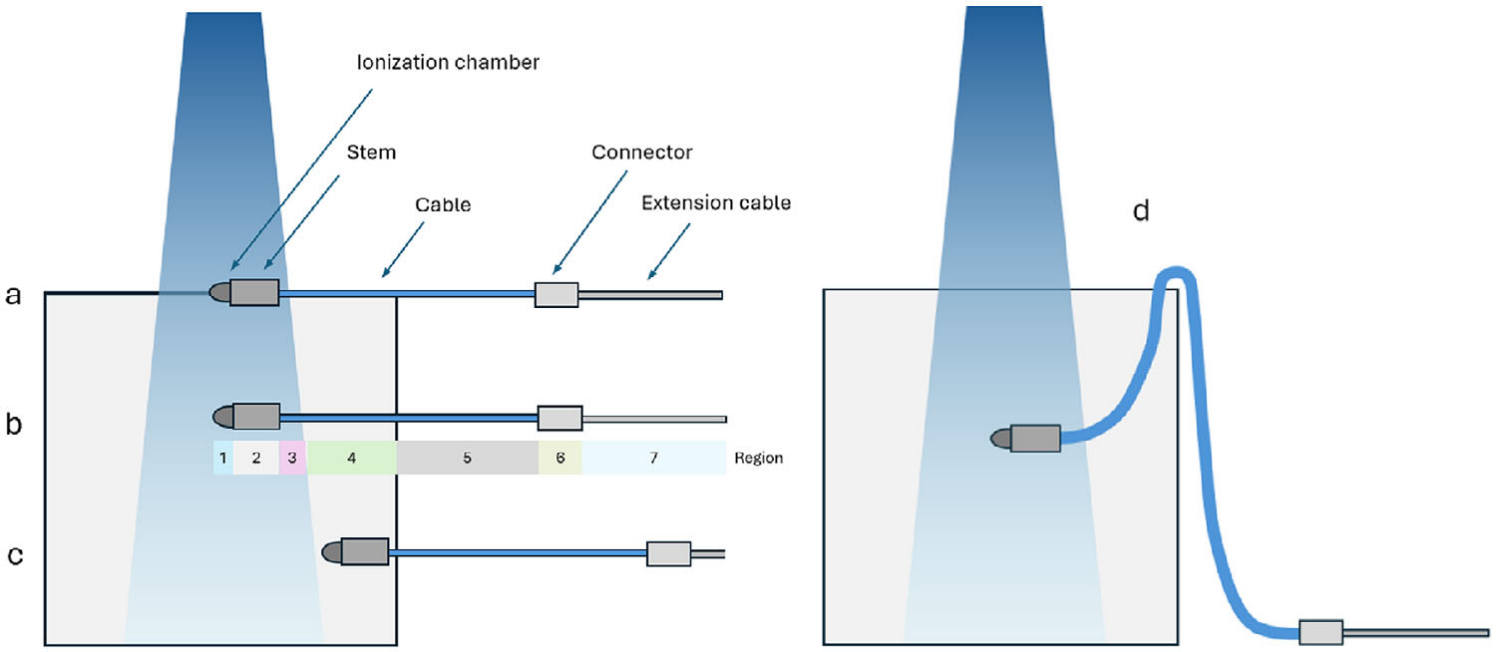
Diagram indicating regions where signal may come from in a typical radiotherapy dosimetry measurement (beam incident from above into water tank). Each region of the detector may be fully or partially exposed to the direct and/or scattered beams (1‐7), and the geometry of each may vary considerably (e.g. an extension cabled coiled next to the phantom, or cable draped over a tank wall). Here a = at surface, b = at depth, c = at depth and out of field, d = with cable draped over tank wall.

There are many regions of an ionization chamber, aside from the ionization volume itself, that may produce a current under irradiation, including the electrodes in the chamber, the stem, the intrinsic cable, the connector and the extension cable. In Figure 10 these are depicted schematically, noting that the radiation field each component encounters depends on the geometry, so that in most cases there are at least 7 distinct regions that could contribute to *M_C_*. The position and length of the cable is not usually controlled with any precision and may change if for example the chamber position changes. Hence measurements of *M_C_* on different setups vary. We also note that the connector may produce a contribution that is opposite in sign to the cable, making it difficult to isolate the source of *M_C_*. With careful measurements and precisely collimated fields it may be possible to isolate and quantify the different components *M*_1‐7_.

## 6 CONCLUSION

We have produced situations with a small volume ionization chamber in large fields where the multiplicative polarity correction takes anomalously large values. We have shown that in our situations the correction is due to a small bias‐independent component of the signal, and a significant part of this comes from the cable. The large values of the polarity correction arise because it is a relative factor, and not necessarily because of effects inside the chamber itself.

We emphasise the importance of measuring the polarity effect, especially when using small volume ionization chambers, large fields and/or novel sources, where significant cable irradiation may occur. Unlike say solid state detectors, ionization chambers are uniquely placed to allow the detection of bias‐independent cable effects. In cases where a large polarity correction is observed, we recommend that authors include details of the ionization current and the cable geometry that may assist in understanding the origin of the correction.

## AUTHOR CONTRIBUTIONS

**Duncan Butler**: Initial Investigation, Methodology, Data Collection, Writing—Original Draft. **Brendan Healy**: Methodology, Supporting Measurements and Confirmation, Interpretation of Data, Writing—Review & Editing.

## CONFLICT OF INTEREST STATEMENT

The authors declare no conflicts of interest.

## Data Availability

Authors will share data upon request to the corresponding author.
